# Supplementary material for: Clueless/CLUH regulates mitochondrial fission by promoting recruitment of Drp1 to mitochondria
Source: Nat Commun. 2022 Mar 24;13:1582. doi: 10.1038/s41467-022-29071-4 (PMC8948191; doi:10.1038/s41467-022-29071-4)
Supplement: Supplementary file 1 — Supplementary Information [file 41467_2022_29071_MOESM1_ESM.pdf]

Supplementary Information

Clueless/CLUH regulates mitochondrial fission by promoting recruitment of Drp1 to mitochondria

Huan Yang[1], Caroline Sibilla[2,3+], Raymond Liu[4‡], Jina Yun[1#], Bruce A. Hay[4], Craig Blackstone[2§], David C. Chan[4], Robert J. Harvey[5,6], Ming Guo[1,7,8*]

[1]Department of Neurology, UCLA David Geffen School of Medicine, Los Angeles, CA, United States; [2]Cell Biology Section, Neurogenetics Branch, National Institute of Neurological Disorders and Stroke, Bethesda, MD, United States; [3]Department of Pharmacology, University College London School of Pharmacy, London, United Kingdom; [4]Division of Biology and Biological Engineering, California Institute of Technology, Pasadena, CA, United States; [5]School of Health and Behavioural Sciences, University of the Sunshine Coast, Queensland, Australia; [6]Sunshine Coast Health Institute, Birtinya, Queensland, Australia; [7]Department of Molecular and Medical Pharmacology, UCLA David Geffen School of Medicine, Los Angeles, CA, United States; [8]California NanoSystems Institute at UCLA, Los Angeles, CA, United States.

*For correspondence: mingfly@ucla.edu

[+]Present address: AstraZeneca PLC, Cambridge Biomedical Campus, Cambridge, United Kingdom.

[‡]Present address: Department of Microbiology and Immunology, UCSF, San Francisco, CA, United States.

[#]Present address: Genentech, Inc., South San Francisco, CA, United States.

[§]Present address: Department of Neurology, Massachusetts General Hospital, Harvard Medical School, Boston, MA, United States.

Supplementary Figures and Figure Legends

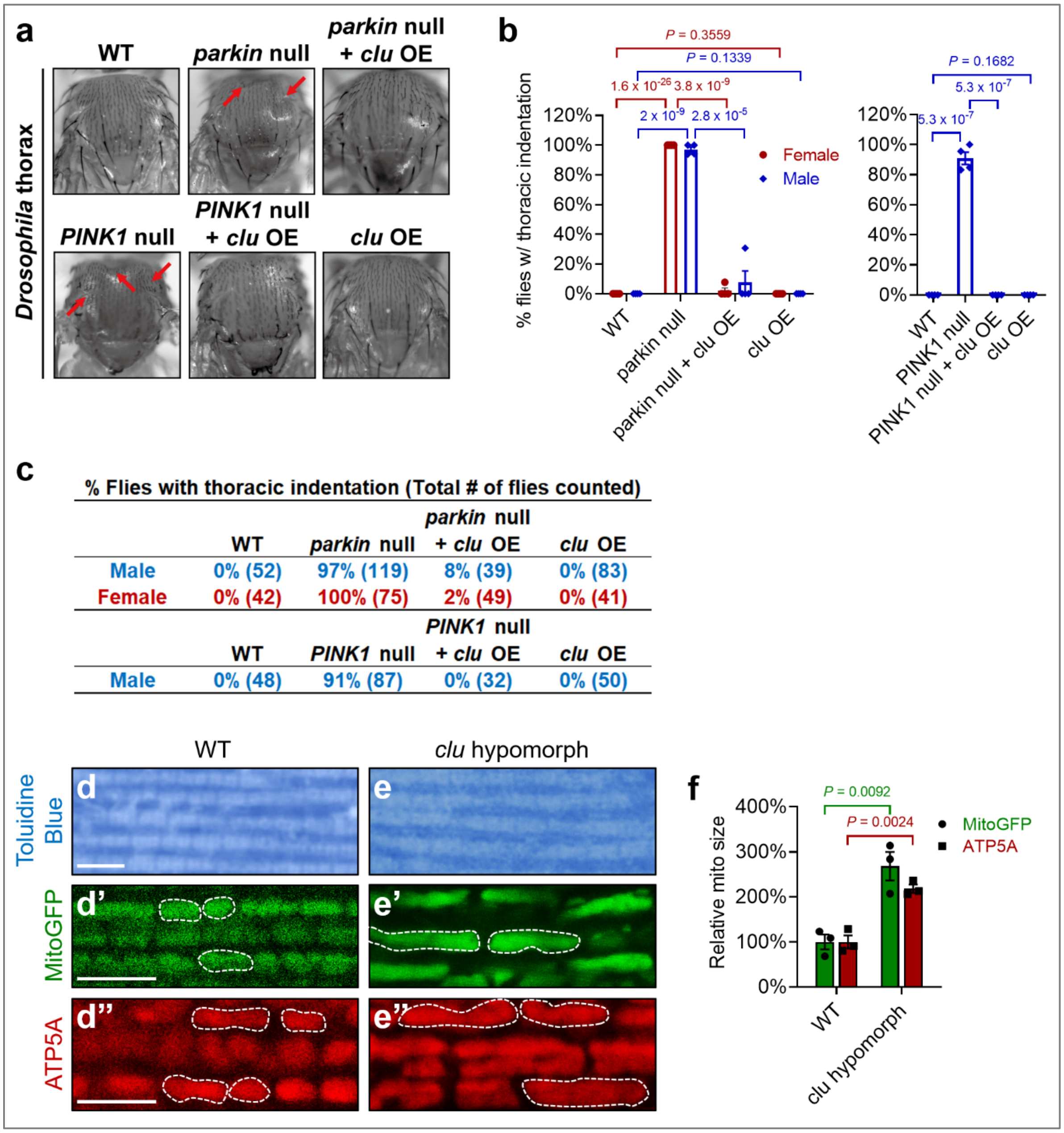

% Flies with thoracic indentation (Total # of flies counted)

	WT	*parkin* null	*parkin* null + *clu* OE	*clu* OE
Male	0% (52)	97% (119)	8% (39)	0% (83)
Female	0% (42)	100% (75)	2% (49)	0% (41)

	WT	*PINK1* null	*PINK1* null + *clu* OE	*clu* OE
Male	0% (48)	91% (87)	0% (32)	0% (50)

Supplementary Figure 1 (for Figure 1) | *clu* overexpression suppresses, and *clu* loss-of-function exacerbates, *PINK1* and *parkin* null mutant phenotypes in *Drosophila*. (a) Images of live *Drosophila* thoraces. Red arrows point to thoracic indentations due to muscle degeneration. Overexpression of *clu* suppresses thoracic indentations in *park*^{25}/*dpk*^{21} null mutants, in addition to those in *PINK1*^{5} null mutants. Expression of UAS-*clu* was driven by Mef2-GAL4. (b) Quantification of flies with thoracic indentations using one-way ANOVA with *post-hoc* Tukey's HSD test, with *P*-values displayed in the graphs. Experiments were repeated 4 times. In each experiment, 5-35 flies were analyzed for each genotype and the average ratio of flies with thoracic indentations was calculated (mean ± SEM, n=4). (c) The average percentage of flies with thoracic indentations is shown, and the total numbers of flies counted in the 4 independent experiments are shown in the parentheses. (d-e'') Characterization of *clu*^{d00713} hypomorphic mutant phenotypes in the muscle. (d-e) Toluidine Blue staining of plastic sections of embedded thoraces shows that *clu*^{d00713} mutants have healthy muscle tissue with no vacuolation. (d'-e', d''-e'') Mitochondria in *clu*^{d00713} mutants are more elongated than those in wildtype flies, as visualized by mitoGFP (d'-e') and by a mouse anti-ATP5A antibody (d''-e''; also Figure 1i, j, o). White dashed lines mark the boundaries of representative mitochondria. Scale bars: 5μM. (f) Mitochondrial size was quantified using Fiji/ImageJ. 50-100 individual mitochondria from 3 different flies for each genotype were analyzed (mean ± SEM, n=3, two-sided Student's *t*-test).

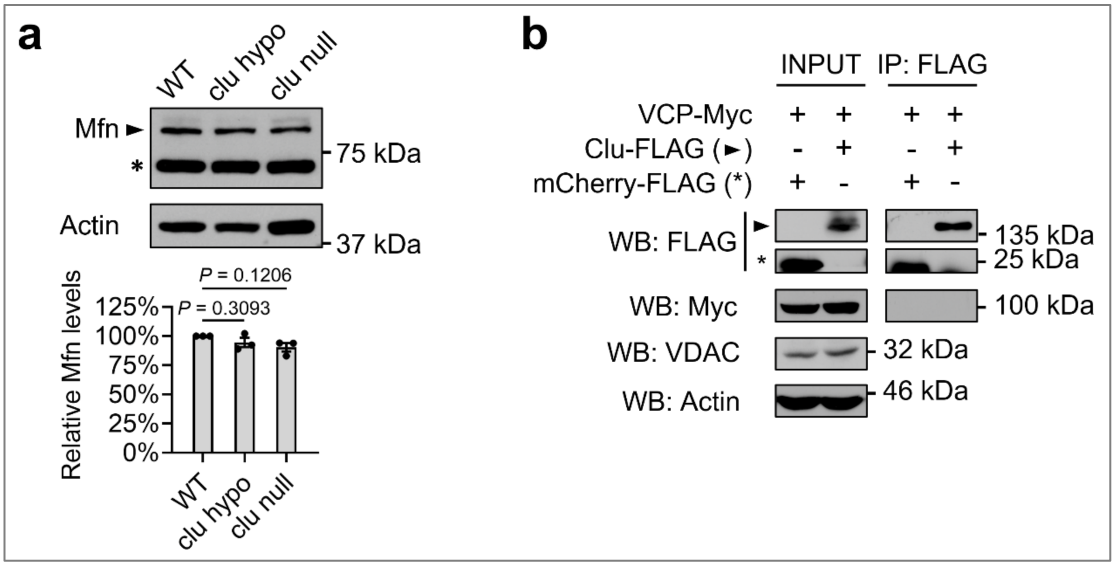

Supplementary Figure 2 (for Figure 4) | Clu does not regulate Mfn levels in *Drosophila*. (a) Western blots and quantification of Mfn levels using *Drosophila* thoracic lysates of the indicated genotypes. Western blots were probed with rabbit anti-Marf (*Drosophila* Mfn) (a generous gift from Dr. Alexander J. Whitworth) and rabbit anti-Actin antibodies, and Mfn levels were normalized to Actin levels. Experiments were performed in triplicate, and representative images are shown. Western blot images were quantified using Fiji/ImageJ, followed by one-way ANOVA with *post-hoc* Tukey's HSD test (mean ± SEM, n=3 independent experiments). (b) Co-IP using lysates from *Drosophila* S2 cells with overexpression of FLAG-tagged Clu and Myc-tagged VCP. The INPUT represents 3% of total lysate to confirm protein expression. VCP-Myc was not co-immunoprecipitated with Clu-FLAG. mCherry-FLAG was used as a control to eliminate any effect caused by the FLAG tag. Co-IP experiments were performed 3 independent times, and representative images are shown. The arrowhead indicates Clu-FLAG and the asterisk indicates the control mCherry-FLAG.

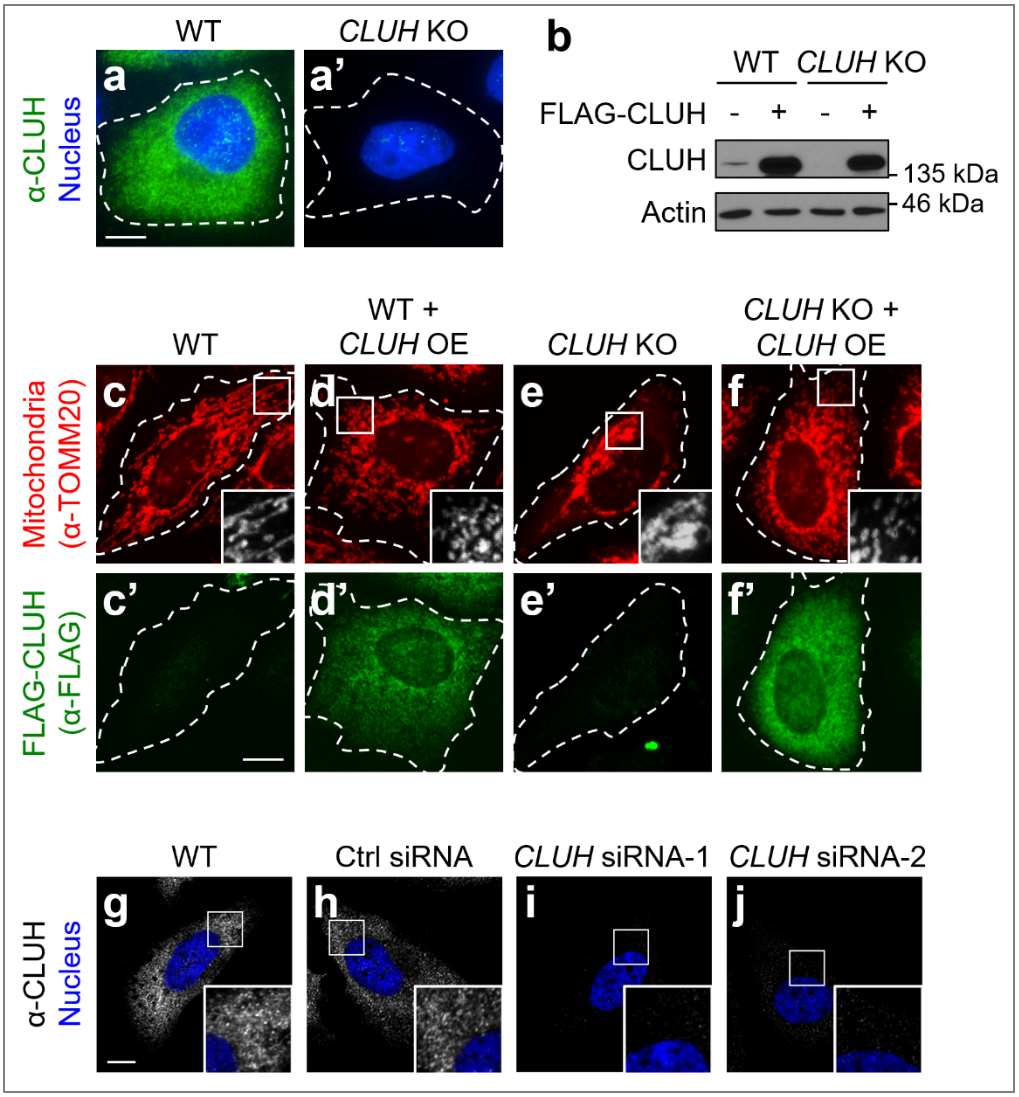

Supplementary Figure 3 (for Figure 5) | CLUH regulates mitochondrial morphology in mammalian cells. (a-a') Immunostaining with a rabbit anti-CLUH antibody confirms absence of CLUH expression in *CLUH* KO (*CLUH-43*) HeLa cells. Experiments were repeated independently for 5 times, and representative images are shown. (b) Western blots were probed with a rabbit anti-CLUH antibody, which confirms *CLUH* KO and *CLUH* overexpression (OE). Experiments were performed 3 independent times, and representative images are shown. (c-f) and (c'-f) are confocal microscopy images of the same cells for each genotype, respectively. (c-f) Mitochondria are labeled with a mouse anti-TOM20 antibody. (c'-f') Cells transfected with a pRK5-FLAG-CLUH plasmid are marked using a rabbit anti-FLAG antibody. Anti-FLAG immunofluorescence signals confirm overexpression of *CLUH* in transfected HeLa cells. Transfection of pRK5-FLAG-CLUH in either wildtype or *CLUH* KO cells results in mitochondrial fragmentation (compare d to c; compare f to e). The insets show enlarged views of the boxed regions (also see Figure 5h-k). In addition, the phonotype of clustered mitochondria shown in *CLUH* KO cells (e; also Figure 5d) is rescued by transfection of pRK5-FLAG-CLUH (f), indicating that this phenotype is specifically due to lack of *CLUH* in the KO cells rather than off-target effects. Experiments were repeated independently for 3 times, and representative images are shown. (g-h) A control siRNA does not change CLUH expression levels or significantly alter mitochondrial morphology (Figure 5h-h', quantified in Figure 5k). (i-j) *CLUH* expression is knocked down using two independent siRNAs, and the knockdown efficiency is confirmed by immunostaining using a rabbit anti-CLUH antibody. (g-j) Experiments were repeated independently for 3 times, and representative images are shown. (a-a', c-f', g-j) Scale bars: 10μM. White dashed lines mark the cell boundaries.

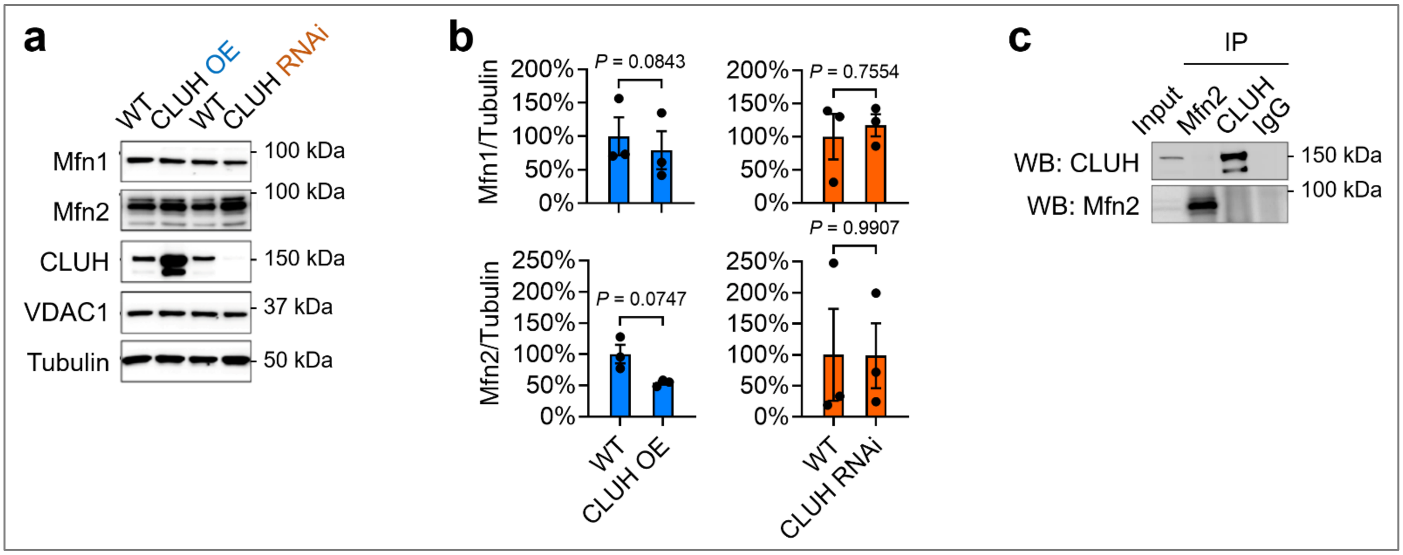

Supplementary Figure 4 (for Figure 6) | *CLUH* does not regulate levels of Mfn1 and Mfn2; CLUH does not bind Mfn2 in mammalian cells. (a-b) Western blots showing Mfn1 and Mfn2 levels in response to *CLUH* OE or *CLUH* RNAi in HeLa cells. Western blot experiments were performed in triplicate, and representative images are shown in (a). Western blot images were quantified using Fiji/ImageJ, and Mfn1 and Mfn2 levels were normalized to α-Tubulin levels (mean ± SEM, n=3 independent experiments, two-sided Student's *t*-test). (c) HeLa cell lysates were subjected to immunoprecipitation with rabbit anti-Mfn2 or rabbit anti-CLUH antibodies, and then immunoblotted with the same antibodies as indicated, to detect interactions between endogenous proteins. Rabbit IgG was used as a negative control. Experiments were performed in triplicate, and representative images are shown.

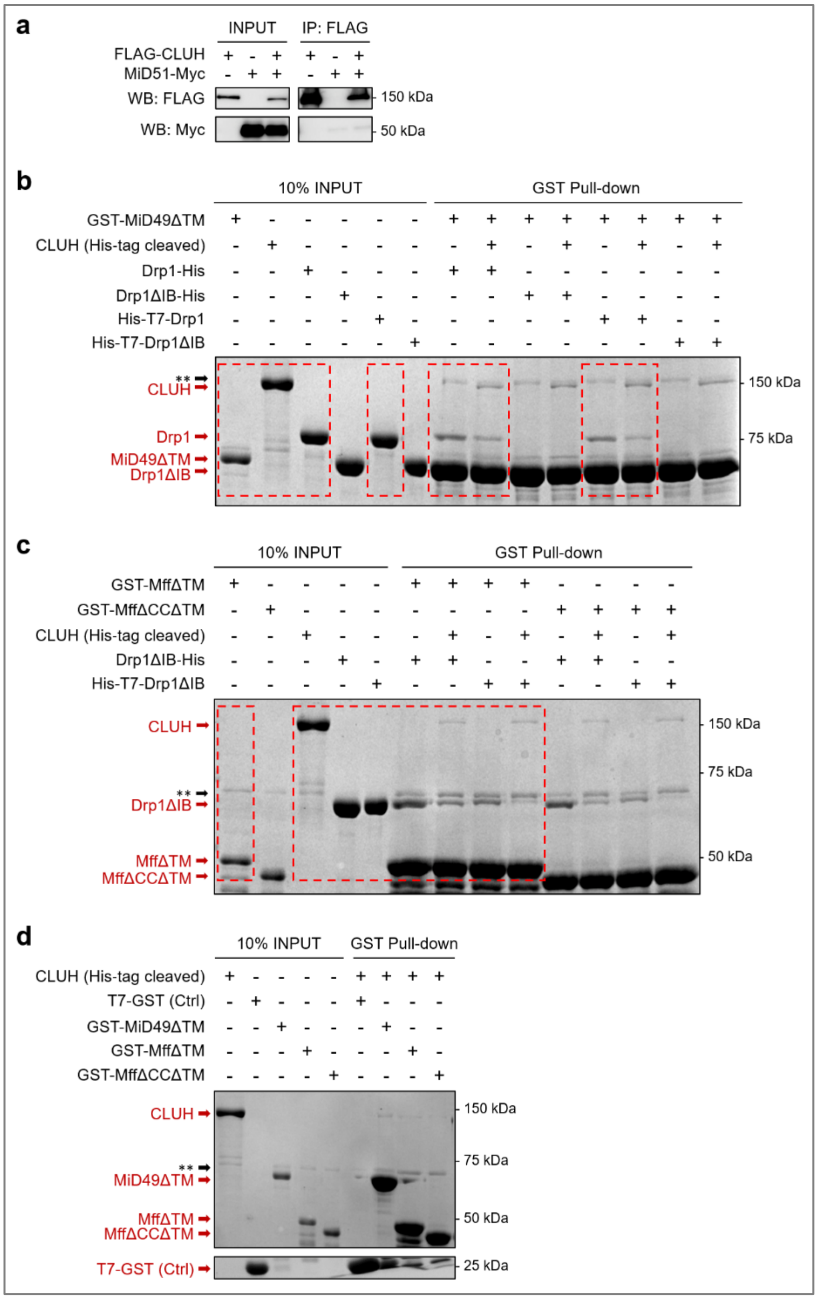

Supplementary Figure 5 (for Figure 7) | Direct protein-protein interactions between CLUH and Drp1 receptors mediate regulation of Drp1 translocation by CLUH. (a) Co-immunoprecipitation experiments show that CLUH binds MiD49 (Figure 7a-b) and Mff (Figure 7c-d) but not MiD51. Experiments were performed in triplicate, and representative images are shown. (b) In a cell-free system with recombinant GST- or His- fusions purified from *E. coli*, MiD49 pulls down both Drp1 and CLUH *in vitro*, in the absence of cross-linkers. CLUH and full-length Drp1 are clearly seen in the respective GST pull-down lanes as indicated. Because Drp1ΔIB has similar molecular weight as MiD49ΔTM, these two proteins cannot be distinguished in the respective GST pull-down lanes. (c) In a parallel experiment performed with purified Mff (isoform-e), CLUH and Drp1 as INPUT, Mff also pulls down both Drp1 and CLUH *in vitro*, without the use of cross-linkers. MffΔTM and MffΔCCΔTM show similar results. Drp1ΔIB was used in this experiment, because removal of the IB domain from Drp1 significantly enhances Drp1-Mff interactions and results in a stable complex *in vitro* (see Results in the main text). (b-c) Drp1 with either an N-terminal or a C-terminal His tag are pulled down at comparable levels. Red dashed lines delineate the boundaries of the parts of the gels shown in Figure 7e-f. (d) In a cell-free system with purified recombinant CLUH and Drp1 receptor proteins, both MiD49 and Mff (isoform-e) pull down CLUH *in vitro*, indicating direct protein-protein interactions. MffΔTM and MffΔCCΔTM show similar results. (b-d) Experiments were repeated independently for 3 times, and representative images are shown. Double asterisks indicate the position of a non-specific band. IB: Insert B domain. TM: transmembrane domain. CC: coiled coil domain.

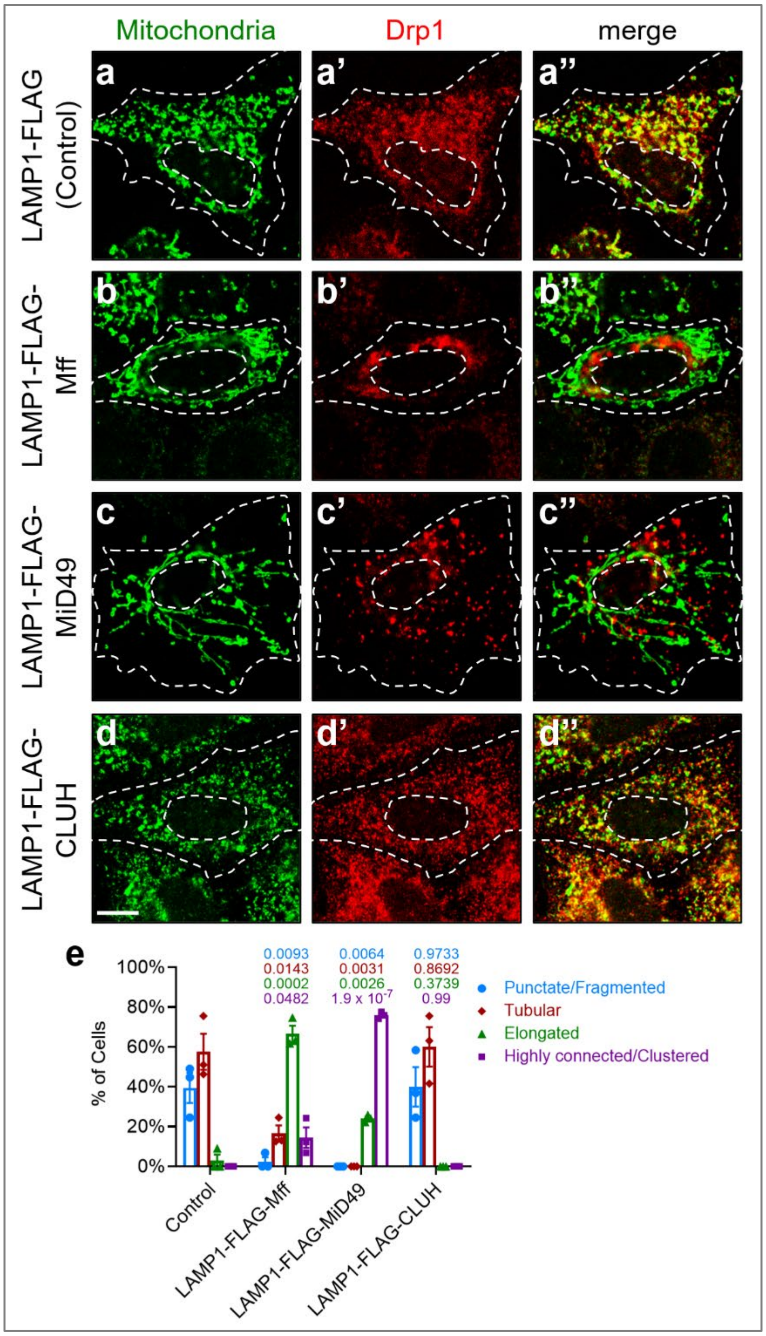

Supplementary Figure 6 (for Figure 7) | Mitochondrial morphology in HeLa cells with expression of lysosome-targeted proteins. (a-d'') The FLAG tag control, or FLAG-tagged Mff, MiD49 or CLUH were ectopically targeted to lysosomes by fusion to LAMP1. Mitochondria are labeled with a goat anti-Hsp60 antibody (green), and Drp1 localization is visualized with a mouse anti-Drp1 antibody (red). As compared to control cells expressing LAMP1-FLAG (a-a''), expression of LAMP1-FLAG-Mff (b-b'') or LAMP-FLAG-MiD49 (c-c') results in significantly elongated or highly connected mitochondria (b, c), because Drp1 proteins are recruited to lysosomes instead of to mitochondria in these cells (b' and c', also see Figure 7k-k'', l-l''). However, expression of LAMP1-FLAG-CLUH does not result in significant changes of mitochondrial morphology (d-d''), because LAMP1-FLAG-CLUH alone is not able to recruit Drp1 to lysosomes (d', also see Figure 7m-m'') and therefore does not disrupt Drp1 recruitment to mitochondria in these cells. Experiments were performed in triplicate, and representative images are shown. White dashed lines mark the boundaries of the cells and the nuclei. Scale bar: 10μM. (e) Mitochondrial morphology in (a-d'') is quantified using the mitochondrial morphology scoring assay (described in Figure 5g). One-way ANOVA with *post-hoc* Tukey's HSD test (mean ± SEM, n=3 independent experiments). In each experiment, >100 cells were analyzed for each genotype, and the percentage of cells in each morphology category was calculated. *P*-values are displayed in the graph. $P < 0.05$: significantly different from the same morphology category in the control group.

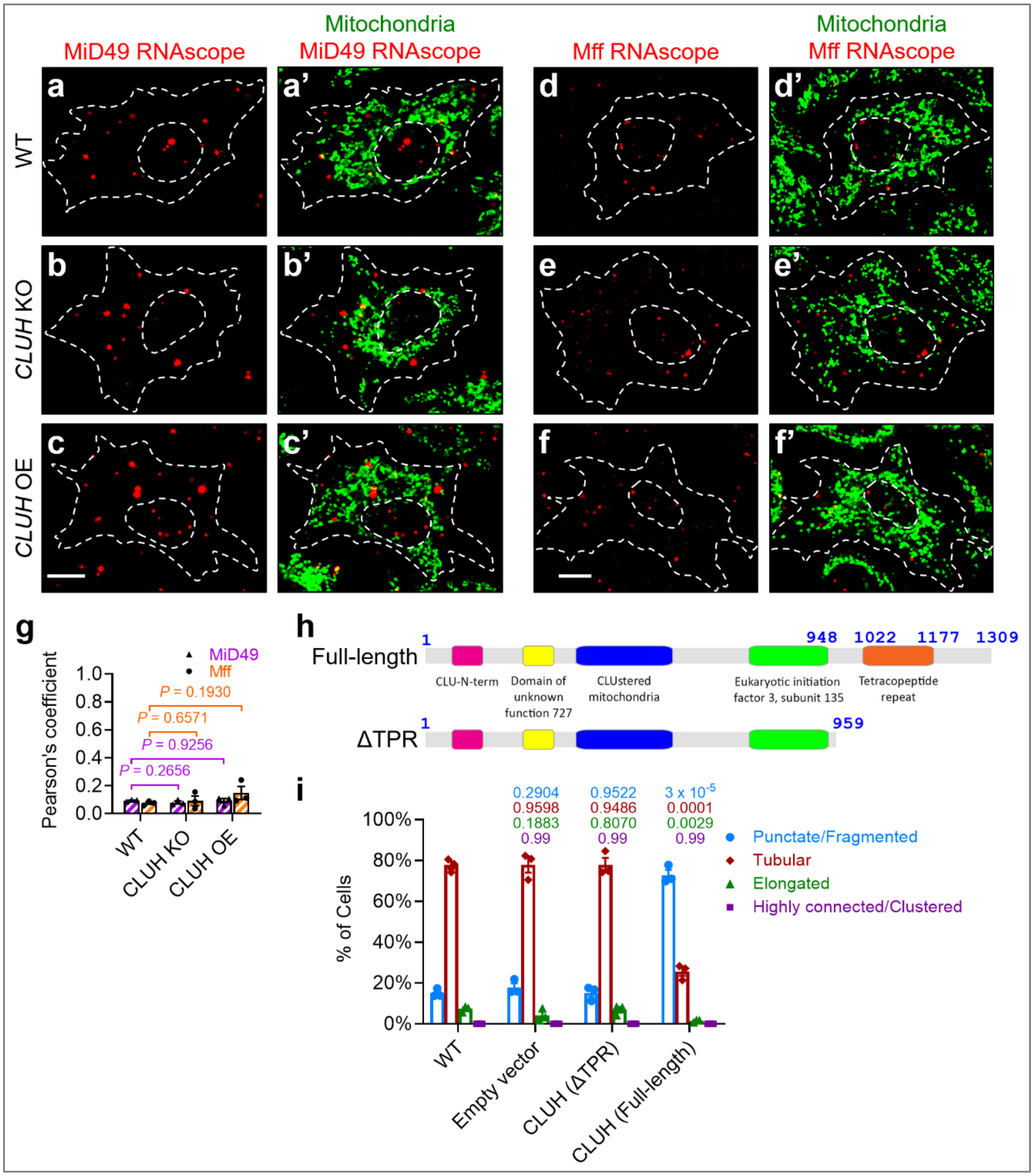

Supplementary Figure 7 (for Figure 8) | CLUH does not regulate localization of Mff/MiD49 mRNAs; the TPR domain, which is essential for RNA binding, is required for the function of CLUH in promoting mitochondrial fission. (a-c') RNAscope assay using probes against *MiD49* mRNAs (red). (d-f') RNAscope assay using probes against *Mff* mRNAs (red). (a-f') An anti-Hsp60 antibody was used at the same time to visualize mitochondria (green). White dashed lines mark the boundaries of the cells and the nuclei. Scale bar: 10μM. (g) Pearson's coefficient indicates co-localization of *MiD49/Mff* mRNAs and mitochondria (mean ± SEM, n=3 independent experiments, one-way ANOVA with *post-hoc* Tukey's HSD test). In each experiment, 10-15 cells were analyzed for each genotype using Fiji/ImageJ, and the average Pearson's coefficient was calculated. (h-i) TPR domain is required for the function of CLUH in promoting mitochondrial fission. (h) Schematics of CLUH domain structure. The numbers indicate the positions of the indicated amino acids (aa). Full-length (1309 aa) or ΔTPR (aa 1-959) CLUH were expressed in HeLa cells by transfection of pRK5-FLAG-CLUH (full-length) or pRK5-FLAG-CLUHΔTPR. (i) Mitochondrial morphology is assessed using the morphology scoring assay (described in Figure 5g). One-way ANOVA with *post-hoc* Tukey's HSD test (mean ± SEM, n=3 independent experiments). In each experiment, >100 cells were analyzed for each genotype, and the percentage of cells in each morphology category was calculated. *P*-values are displayed in the graph. $P < 0.05$: significantly different from the same morphology category in the wildtype group (WT, non-transfected cells).

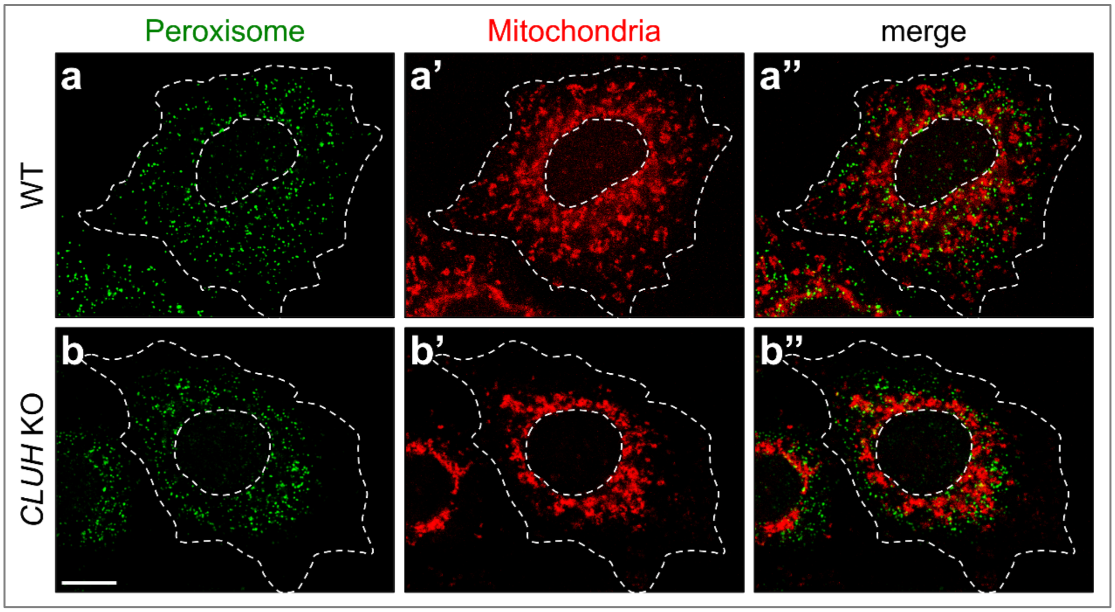

Supplementary Figure 8 | CLUH does not regulate peroxisomal morphology in mammalian cells. (a, a’, a’’) Confocal microscopy images of the same wildtype HeLa cell. (b, b’, b’’) Confocal microscopy images of the same *CLUH* KO HeLa cell. (a, b) Peroxisomes are labeled with a rabbit anti-PMP70 antibody (green). (a’, b’) Mitochondria are labeled with a mouse anti-TOM20 antibody (red). No significant morphological changes of peroxisomes were observed in *CLUH* KO cells (b) as compared to those in wildtype cells (a). Experiments were repeated independently for 3 times, and representative images are shown. White dashed lines mark the boundaries of the cells and the nuclei. Scale bar: 10μM.

Supplementary Tables

	Day with 50% survival	Day with 25% survival	Day with 5% survival	Total # of flies counted	Genotype
**WT**	72	82	122	233	*w; Mef2-GAL4/+*
***clu* null**	3.5	3.5	4.5	103	*w; clu*^{f04554}
***clu* null + Mef2-GAL4**	3.5	4.5	6.5	92	*w; clu*^{f04554} *; Mef2-GAL4/+*
clu* null + UAS-*drp1	3.5	4	5.5	72	*w; clu*^{f04554} *; UAS-drp1/+*
***clu* null + *drp1* OE**	7	11.5	19	96	*w; clu*^{f04554} *; Mef2-GAL4/UAS-drp1*

Supplementary Table 1 (for Figure 3) | Overexpression of *drp1* suppresses adult lethality of *clu*^{f04554} null mutants in *Drosophila*. Details of the longevity assay is discussed in Figure 3a. Days with 50%, 25% and 5% survival for flies of the indicated genotypes, and total number of flies counted are shown here. Data was collected from the longevity assay using male flies. Experiments were repeated in triplicate with consistent results. Parallel experiments using female flies showed the same results.

Genotype	*clu* null	*clu* null + Mef2-GAL4		*clu* null + UAS-*drp1*		*clu* null + *drp1* OE	
	w; clu^{f04554}	*w; clu*^{f04554} *; Mef2-GAL4/+*		*w; clu*^{f04554} *; UAS-drp1/+*		*w; clu*^{f04554} *; Mef2-GAL4/UAS-drp1*	
**Day**	**% Survival**	**% Survival**	**P-value**	**% Survival**	**P-value**	**% Survival**	**P-value**
**1**	100%	100%	_	100%	_	100%	_
**2**	96%	99%	0.596	97%	0.887	98%	0.749
**3**	65%	85%	0.367	69%	0.759	82%	0.442
**4**	17%	37%	0.099	25%	0.488	75%	0.006
**5**	4%	11%	0.415	13%	0.124	70%	0.0002
**6**	1%	7%	0.365	3%	0.668	53%	0.046
**7**	0%	4%	0.201	0%	_	50%	0.028
**8**	0%	1%	0.423	0%	_	40%	0.016
**9**	0%	0%	_	0%	_	30%	0.025
**10**	0%	0%	_	0%	_	29%	0.017
**11**	0%	0%	_	0%	_	27%	0.005
**12**	0%	0%	_	0%	_	23%	0.036
**13**	0%	0%	_	0%	_	22%	0.024
**14**	0%	0%	_	0%	_	17%	0.002
**15**	0%	0%	_	0%	_	11%	0.029
**16**	0%	0%	_	0%	_	10%	0.007
**17**	0%	0%	_	0%	_	8%	0.092
**18**	0%	0%	_	0%	_	7%	0.180
**19**	0%	0%	_	0%	_	5%	0.264
**20**	0%	0%	_	0%	_	2%	0.423
**21**	0%	0%	_	0%	_	2%	0.423
**22**	0%	0%	_	0%	_	2%	0.423
**23**	0%	0%	_	0%	_	2%	0.423
**24**	0%	0%	_	0%	_	1%	0.423
**25**	0%	0%	_	0%	_	1%	0.423
**26**	0%	0%	_	0%	_	0%	_

Supplementary Table 2 (for Figure 3) | Overexpression of *drp1* suppresses the adult lethality of *clu*^{f04554} null mutants in *Drosophila*. Details of the longevity assay is discussed in Figure 3b. Statistical analysis of day-to-day survival rate for flies of the indicated genotypes is shown here. The days with live flies remaining are highlighted in a light green background. Survival rate of the indicated genotypes was compared to that of *clu*^{f04554} mutants on each day (One-way ANOVA with *post-hoc* Tukey's HSD test). Each comparison with a significant difference ($P < 0.05$) is highlighted in a blue background, with $0.01 < P < 0.05$ shown in light blue, $0.001 < P < 0.01$ in medium blue, and $P < 0.001$ in dark blue. Those without highlights have a *P*-value bigger than 0.05 (not significant). Data in this Table was collected using male flies. Experiments were repeated in triplicate with consistent results (mean ± SEM, n=3 independent experiments). In total, 72-233 male flies were collected for each genotype (Supplementary Table 1). These flies were monitored throughout the lifespan and survival rate was calculated on a daily basis. Parallel experiments using female flies showed the same results.

Gene	GenBank Accession No.	Vendor
CLUH (EST1)	AA765709	imaGenes, UK
CLUH (EST2)	CR627449	imaGenes, UK
MiD51	BC008327, BE312328	Thermo Fisher Scientific, USA

Supplementary Table 3 (for Methods - Molecular cloning for mammalian cell experiments) | EST information. The EST clones used for PCR amplification to obtain full-length *CLUH* and *MiD51* DNA sequences, which were then subcloned into the pRK5-FLAG and pRK5-Myc vectors, respectively.

	siRNA oligonucleotide sequence	Nucleotide positions in the *CLUH* gene
siRNA1	CCCGCTACCTCATGCTGCTGGTGTT	3,263 - 3,287
siRNA2	TCCTTCCTCCTTGGAGTCCAAGTCT	1,938 – 1,962

Supplementary Table 4 (for Methods - RNAi in mammalian cells) | siRNA sequences. Two stealth siRNAs used to knock down *CLUH* in HeLa cells.

	DNA sequence	Spacer direction
Spacer-1	GAAAATGGGCTTGACGAGGC	Sense
Spacer-2	GGTGGTCTCATCTCCCGGGC	Anti-sense
Spacer-3	GACAATGACTTCCTGGCCGG	Anti-sense
Spacer-4	GGGAGATGAGACCACCGGCC	Sense
Genomic DNA PCR Forward Primer	GGTTCAGGTGCAAGCCATAC	_
Genomic DNA PCR Reverse Primer	CTCTGCCCTTGGAGATAACC	_
Genomic DNA Sequencing Forward Primer	TGCCTGATTCTGACCCGTTC	_

Supplementary Table 5 (for Methods - Generation of *CLUH* knockout HeLa cells using the CRISPR-Cas9 system) | Sequences of spacers and primers used in generating *CLUH* knockout HeLa cells. Detailed experimental procedure is described in the Methods section.
