# Supplementary material for: Clueless/CLUH regulates mitochondrial fission by promoting recruitment of Drp1 to mitochondria
Source: Nat Commun. 2022 Mar 24;13:1582. doi: 10.1038/s41467-022-29071-4 (PMC8948191; doi:10.1038/s41467-022-29071-4)
Supplement: Supplementary file 3 — Reporting Summary [file 41467_2022_29071_MOESM3_ESM.pdf]

nature research

Corresponding author(s): Ming Guo

Last updated by author(s): Dec 23, 2021

Reporting Summary

Nature Research wishes to improve the reproducibility of the work that we publish. This form provides structure for consistency and transparency in reporting. For further information on Nature Research policies, see our Editorial Policies and the Editorial Policy Checklist.

Statistics

For all statistical analyses, confirm that the following items are present in the figure legend, table legend, main text, or Methods section.

n/a	Confirmed	
☐	☒	The exact sample size (*n*) for each experimental group/condition, given as a discrete number and unit of measurement
☐	☒	A statement on whether measurements were taken from distinct samples or whether the same sample was measured repeatedly
☐	☒	The statistical test(s) used AND whether they are one- or two-sided<br>*Only common tests should be described solely by name; describe more complex techniques in the Methods section.*
☒	☐	A description of all covariates tested
☐	☒	A description of any assumptions or corrections, such as tests of normality and adjustment for multiple comparisons
☐	☒	A full description of the statistical parameters including central tendency (e.g. means) or other basic estimates (e.g. regression coefficient) AND variation (e.g. standard deviation) or associated estimates of uncertainty (e.g. confidence intervals)
☐	☒	For null hypothesis testing, the test statistic (e.g. F, t, r) with confidence intervals, effect sizes, degrees of freedom and P value noted<br>*Give P values as exact values whenever suitable.*
☒	☐	For Bayesian analysis, information on the choice of priors and Markov chain Monte Carlo settings
☒	☐	For hierarchical and complex designs, identification of the appropriate level for tests and full reporting of outcomes
☐	☒	Estimates of effect sizes (e.g. Cohen's *d*, Pearson's *r*), indicating how they were calculated

Our web collection on statistics for biologists contains articles on many of the points above.

Software and code

Policy information about availability of computer code

Data collection	Zeiss ZEN software (black edition) for the confocal microscope, KODAK Molecular Imaging software for the KODAK Gel Logic 112 imaging system.
Data analysis	Fiji/Image J v2.0.0-rc-689/1.52e, GraphPad Prism v.9.2.0 , Microsoft Excel (Version 2106), Bio-Rad CFX Maestro 1.1.

For manuscripts utilizing custom algorithms or software that are central to the research but not yet described in published literature, software must be made available to editors and reviewers. We strongly encourage code deposition in a community repository (e.g. GitHub). See the Nature Research guidelines for submitting code & software for further information.

Data

Policy information about availability of data

All manuscripts must include a data availability statement. This statement should provide the following information, where applicable:

- Accession codes, unique identifiers, or web links for publicly available datasets
- A list of figures that have associated raw data
- A description of any restrictions on data availability

All data are available within the Article, Supplementary Information or Source Data file. Source data for Figure 3a-b and Supplementary Table 1 have been provided as Supplementary Table 2. All other source data are provided in the Source Data file with this paper. The knockout cell lines and transgenic flies generated in this work are available from the corresponding author upon request.

Field-specific reporting

Please select the one below that is the best fit for your research. If you are not sure, read the appropriate sections before making your selection.

☒ Life sciences ☐ Behavioural & social sciences ☐ Ecological, evolutionary & environmental sciences

For a reference copy of the document with all sections, see nature.com/documents/nr-reporting-summary-flat.pdf

Life sciences study design

All studies must disclose on these points even when the disclosure is negative.

Sample size	No statistical methods were used to predetermine the sample size. Sample size was determined to be adequate based on previously published researches. All sample size, statistical tests and P-values are indicated in the Figures and Legends, as well as described in the Methods. Phenotypes observed did not vary dramatically within each analyzed group or among biological replicates.<br><br>Drosophila experiments: Confocal microscope imaging of Drosophila tissue (MitoGFP, TUNEL and immunohistochemistry experiments) was carried out with at least 3 biological replicates. At least 3 individual flies were used per genotype. Toluidine blue staining and TEM analysis were carried out using plastic sections of embedded individual Drosophila thorax, and the number of biological replicates for each genotype was at least 3. To quantify the number of flies having thoracic indentation (Supplementary Figure 1a-b), experiments were performed in quadruplicate, and a total number of 32 to 119 flies were counted for each genotype, as indicated in the Figures and Legends. ATP assay was performed in triplicate, and 6 flies were used per genotype per biological replicate. To quantify mitochondrial morphology in Drosophila muscle, 50 to 100 individual mitochondria from 3 different flies were analyzed for each genotype. Drosophila longevity assay (Figure 3a-b, Supplementary Tables 1-2) was performed in triplicate, with a total number of 72 to 233 flies counted for each genotype, as indicated in the Figures and Legends.<br><br>Mammalian cell experiments: To quantify mitochondrial morphology in HeLa cells, experiments were performed in triplicate. A mitochondrial morphology scoring assay (described in Figure 5g) was used, with a total number of >200 cells counted per genotype per biological replicate. Confocal microscope imaging of cells (immunocytochemistry experiments, RNAscope, PLA and Puro-PLA) was carried out with at least 3 biological replicates. In each independent experiment, >10 cells were analyzed, and the average was calculated and displayed as one data point in the dot plot, as indicated in the Figures and Legends.
Data exclusions	No data were excluded from the analyses.
Replication	All the experiments in this study were performed with at least 3 independent biological replicates, and experimental findings were reliably reproduced. Imaging of Drosophila tissue using confocal microscope, light microscope or TEM was carried out with at least 3 biological replicates. Assays based on cultured mammalian cells were carried out and repeated with 3-4 independent experiments. Immunoprecipitation, Western blotting, and in vitro pull-down experiments were performed with at least 3 biological replicates. RT-PCR and qPCR experiments were performed with 3 biological replicates.
Randomization	Experimental groups were formed based on genotypes. For in vivo experiments, flies were randomly selected within each experimental group. For confocal microscope, light microscope or TEM imaging, multiple regions of interest (ROI) were randomly assigned for image acquisition within each experimental group.
Blinding	For data analysis, blinding was not possible because (1) the same researcher set up the experiments and analysed the data; (2) in the presence of mitochondrial markers in the images, experimental group showed significant changes to mitochondrial morphology as compared to the control group. Unbiased experimental procedure and data analysis were carried out wherever possible.

Reporting for specific materials, systems and methods

We require information from authors about some types of materials, experimental systems and methods used in many studies. Here, indicate whether each material, system or method listed is relevant to your study. If you are not sure if a list item applies to your research, read the appropriate section before selecting a response.

Materials & experimental systems

n/a	Involved in the study	
☐	☒	Antibodies
☐	☒	Eukaryotic cell lines
☒	☐	Palaeontology and archaeology
☐	☒	Animals and other organisms
☒	☐	Human research participants
☒	☐	Clinical data
☒	☐	Dual use research of concern

Methods

n/a	Involved in the study	
☒	☐	ChIP-seq
☒	☐	Flow cytometry
☒	☐	MRI-based neuroimaging

April 2020

Antibodies

Antibodies used

Primary antibodies used:
mouse anti-HA (Millipore, Cat. # 05-904, Lot # 3045580),
rabbit anti-HA (MilliporeSigma/Sigma-Aldrich, H6908),
mouse anti-Myc (Millipore, clone 4A6, Cat. # 05-724),
rabbit anti-Myc (Cell Signaling Technology, 71D10, Product # 2278),
mouse anti-FLAG (GenScript Biotech, clone 5A8E5, Cat. # A00187),
mouse anti-FLAG (MilliporeSigma/Sigma-Aldrich, clone M2, Product # F1804),
rabbit anti-FLAG (GenScript Biotech, Cat. # A01868),
mouse anti-GFP (Sigma-Aldrich, clone GFP-20, ascites fluid, G6539),
rabbit anti-GFP (Thermo Fisher Scientific, Cat. # A-11122),
rabbit anti-CLUH/eIF3X (Bethyl laboratories, A301-765A),
rabbit anti-CLUH (Aviva Systems Biology, ARP70642_P050),
mouse anti-Drp1 (Abcam, ab56788),
rabbit anti-Marf (a gift from Dr. Alexander J. Whitworth),
mouse anti-Mfn1 + Mfn2 antibody (Abcam, ab57602),
rabbit anti-Mfn2 (Proteintech Group, Inc, Cat. # 12186-1-AP),
mouse anti-Porin (MitoSciences Inc., MSA03/Abcam, ab14734),
rabbit anti-VDAC1/Porin (Abcam, ab34726),
rabbit anti-Actin (MilliporeSigma/Sigma-Aldrich, A2066),
mouse anti-α-Tubulin (MilliporeSigma/Sigma-Aldrich, T5168),
mouse anti-ATP5A (MitoSciences Inc., MS507/Abcam, ab14748),
mouse anti-TOM20 (BD Transduction Laboratories, Cat. # 612278),
goat anti-Hsp60 (Santa Cruz Biotechnology, sc-1052),
mouse anti-Puromycin (Kerafast, 3RH11),
rabbit anti-Mff (Proteintech, 17090-1-AP),
rabbit anti-MiD49/SMCR7 (Thermo Fisher Scientific, PA5-99984).

Secondary antibodies used:
ECL anti-rabbit IgG HRP-linked whole antibody from donkey (GE Healthcare, Cat. # NA9340V),
ECL anti-mouse IgG HRP-linked whole antibody from sheep (GE Healthcare, Cat. # NXA931),
Alexa Fluor 488 goat anti-rabbit IgG (Thermo Fisher Scientific, Cat. # A-11034),
Alexa Fluor 488 goat anti-mouse IgG (Thermo Fisher Scientific, Cat. # A-11029),
Alexa Fluor 488 donkey anti-rabbit IgG (Thermo Fisher Scientific, Cat. # A-21206),
Alexa Fluor 488 donkey anti-mouse IgG (Thermo Fisher Scientific, Cat. # A-21202),
Alexa Fluor 488 donkey anti-goat IgG (Thermo Fisher Scientific, Cat. # A-11055),
Alexa Fluor 546 goat anti-rabbit IgG (Thermo Fisher Scientific, Cat. # A-11035),
Alexa Fluor 546 donkey anti-mouse IgG (Thermo Fisher Scientific, Cat. # A-10036),
Alexa Fluor Plus 594 donkey anti-goat IgG (Thermo Fisher Scientific, Cat. # A32758).

Validation

The rabbit anti-Marf (Drosophila Mfn) antibody was a gift from Dr. Alexander J. Whitworth. Please see Ziviani, Tao and Whitworth, PNAS (2010) for validation.

All other primary antibodies were commercially obtained and validated by the manufacturer, with detailed validation analysis and relevant literatures provided on the company website:
mouse anti-HA (Millipore, https://www.emdmillipore.com/US/en/product/Anti-HA-Tag-Antibody,MM_NF-05-904),
rabbit anti-HA (MilliporeSigma/Sigma-Aldrich, https://www.sigmaaldrich.com/US/en/product/sigma/h6908),
mouse anti-Myc (Millipore, https://www.emdmillipore.com/US/en/product/Anti-Myc-Tag-Antibody-clone-4A6,MM_NF-05-724),
rabbit anti-Myc (Cell Signaling Technology, https://www.cellsignal.com/products/primary-antibodies/myc-tag-71d10-rabbit-mab/2278),
mouse anti-FLAG (GenScript Biotech, https://www.genscript.com/antibody/A00187-THE_DYKDDDDK_Tag_Antibody_mAb_Mouse.html),
mouse anti-FLAG (MilliporeSigma/Sigma-Aldrich, https://www.sigmaaldrich.com/US/en/product/sigma/f1804),
rabbit anti-FLAG (GenScript Biotech, https://www.genscript.com/antibody/A01868-MonoRab_DYKDDDDK_Tag_Antibody_mAb_Rabbit.html),
mouse anti-GFP (Sigma-Aldrich, https://www.sigmaaldrich.com/US/en/product/sigma/g6539),
rabbit anti-GFP (Thermo Fisher Scientific, https://www.thermofisher.com/antibody/product/GFP-Antibody-Polyclonal/A-11122),
rabbit anti-CLUH/eIF3X (Bethyl laboratories, https://www.thermofisher.com/antibody/product/eIF3X-Antibody-Polyclonal/A301-765A),
rabbit anti-CLUH (Aviva Systems Biology, https://www.avivasysbio.com/cluh-antibody-n-terminal-region-arp70642-p050.html),
mouse anti-Drp1 (Abcam, https://www.abcam.com/drp1-antibody-3b5-ab56788.html),
mouse anti-Mfn1 + Mfn2 antibody (Abcam, https://www.abcam.com/mitofusin-2--mitofusin-1-antibody-3c9-ab57602.html),
rabbit anti-Mfn2 (Proteintech Group, Inc, https://www.ptglab.com/products/MFN2-Antibody-12186-1-AP.htm),
mouse anti-Porin (MitoSciences Inc., https://www.abcam.com/vdac1porin--vdac3-antibody-20b12af2-ab14734.html),
rabbit anti-VDAC1/Porin (Abcam, https://www.abcam.com/vdac1porin-antibody-ab34726.html),
rabbit anti-Actin (MilliporeSigma, https://www.sigmaaldrich.com/US/en/product/sigma/a2066),
mouse anti-α-Tubulin (MilliporeSigma, https://www.sigmaaldrich.com/US/en/product/sigma/t5168),
mouse anti-ATP5A (MitoSciences/Abcam, https://www.abcam.com/atp5a-antibody-15h4c4-mitochondrial-marker-ab14748.html),
mouse anti-TOM20 (BD Transduction Laboratories, https://www.bdbiosciences.com/en-us/products/reagents/microscopy-imaging-reagents/immunofluorescence-reagents/purified-mouse-anti-tom20.612278),
goat anti-Hsp60 (Santa Cruz Biotechnology, https://www.scbt.com/p/hsp-60-antibody-n-20),
mouse anti-Puromycin (Kerafast, https://www.kerafast.com/productgroup/190/anti-puromycin-3rh11-antibody),
rabbit anti-Mff (Proteintech, https://www.ptglab.com/products/MFF-Antibody-17090-1-AP.htm),

rabbit anti-MiD49/SMCR7 (Thermo Fisher Scientific, https://www.thermofisher.com/antibody/product/SMCR7-Antibody-Polyclonal/PA5-99984).

Eukaryotic cell lines

Policy information about cell lines

Cell line source(s)	HeLa (wildtype): a gift from Dr. Richard J. Youle, which was originally acquired from the ATCC.<br>HeLa (CLUH KO-40 and CLUH KO-43): two CLUH KO cell lines generated in this study, using the above wildtype HeLa cells as background and the CRISPR-Cas9 system for gene editing.
Authentication	HeLa (wildtype) cell line was a gift from Dr. Richard J. Youle. This HeLa cell line was originally acquired from the ATCC and authenticated by the Johns Hopkins GRCF Fragment Analysis Facility using STR profiling. Please see Narendra, Tanaka, Suen and Youle, J Cell Biol (2008) and Lazarou et al, Nature (2015) for authentication. The two CLUH KO HeLa cell lines (CLUH KO-40 and CLUH KO-43) were generated in this study using the CRISPR-Cas9 system. Both KO lines were characterized using PCR of genomic DNA, and the absence of CLUH in both KO lines was confirmed using Western blotting and immunostaining with an anti-CLUH antibody.
Mycoplasma contamination	We confirmed that cell lines were free of mycoplasma.
Commonly misidentified lines<br>(See ICLAC register)	No commonly misidentified cell lines were used in this study.

Animals and other organisms

Policy information about studies involving animals; ARRIVE guidelines recommended for reporting animal research

Laboratory animals	The Drosophila melanogaster strains used in this study were obtained from the following sources:<br>The CaSpeR-FLAG-FlAsH-HA-drp1 strain was a gift from Dr. Hugo J. Bellen. To generate UAS-clueless (clu) and UAS-FLAG-clu transgenic flies, PTW/PTWF-clu constructs were injected into w1118 flies (Rainbow Transgenic Flies, Inc, Camarillo, CA, USA). Multiple independent fly strains were collected and analyzed, and overexpression of clu was confirmed with RT-qPCR and Western blotting. The clu[f04554] null mutant strain and the clu[d00713] hypomorph mutant strain were obtained from the Exelixis Collection at the Harvard Medical School. The UAS-clu RNAi strains were obtained from Vienna Drosophila Resource Center (VDRC). PINK1[5], parkin[25], dpk21, UAS-drp1, UAS-mfn RNAi, IFM-GAL4 and Mef2-GAL4 flies were generated/used in our previous work and are described in Clark et al., Nature (2006), Deng et al., PNAS (2008), Yun et al., eLife (2014). The yw, hsFLP70 (BDSC #6420) and yw; neoFRT42D (BDSC #5616) strains were obtained from the Bloomington Drosophila Stock Center (BDSC). Transgenic flies were balanced against the same genetic background: w1118; Bl/CyO; TM2/TM6B.<br><br>Experiments were performed using both male and female flies with consistent results. In each independent experiment, only male flies were used or only female flies were used. For thoracic indentation scoring, MitoGFP, immunohistochemistry, Toluidine blue staining, TEM experiments and ATP assay, 2-day-old flies were used. For TUNEL assays, 7-day-old (Figure 1) and 2-day-old (Figure 3) flies were used. clu[f04554] null mutants show adult lethality (dying 3-6 days after eclosure), so all Drosophila histology experiments using clu[f04554] null mutant flies were done with flies that were no more than 3-day-old. For longevity assays, flies were monitored throughout their lifespan (wildtype: ~120 days).
Wild animals	This study did not involve wild animals.
Field-collected samples	This study did not involve field-collected samples.
Ethics oversight	Drosophila strains were maintained in a 25°C humidified incubator. Drosophila strains were raised and handled according to standard institutional regulations.

Note that full information on the approval of the study protocol must also be provided in the manuscript.
